# Duplicated *Pax6* Gene Expression During Nervous System Development in the Asexually Reproducing Annelid *Nais communis*

**DOI:** 10.3390/biology14121704

**Published:** 2025-11-29

**Authors:** Roman P. Kostyuchenko, Aleksandr I. Kotenko, Ekaterina A. Checheneva

**Affiliations:** Department of Embryology, St. Petersburg State University, Universitetskaya nab. 7-9, 199034 St. Petersburg, Russia; sasha.tele@gmail.com (A.I.K.); katblack2605@gmail.com (E.A.C.)

**Keywords:** *Pax6*, gene expression, architecture of the nervous system, nervous system development, tissue remodeling, asexual reproduction, *Nais communis*, Annelida, evolution

## Abstract

*Pax6* plays a highly conserved role in the formation of the eye, development, and patterning of the nervous system across bilaterians. Nevertheless, there are no studies focusing on the role of *Pax6* during asexual reproduction, a developmental trajectory that is widespread among metazoans. The present study represents the first investigation of *Pax6* gene expression during agametic propagation in annelids. We identified in the asexually reproducing annelid *Nais communis* four homologs of *Pax6* and examined their developmental patterns by in situ hybridization. To establish a morphological basis for the expression patterns, we used immunohistochemistry and confocal laser scanning microscopy to describe the nervous system architecture of the growing adults and remodeling of the original ventral nerve cord, development of the new brain, ventral ganglia, peripheral nerves, and sensory organs in asexually reproducing worms. Our results support the hypothesis of an evolutionarily conserved function of *Pax6* genes in the development of the eye and other sensory organs, as well as the central nervous system, among bilaterians, regardless of developmental trajectory. On the other hand, identified *Pax6* homologs show differential expression within the developing new head and tail ends. Differences in spatiotemporal expression patterns may be evidence of functional diversification of duplicated homologs.

## Simple Summary

During asexual reproduction by paratomic fission, the new head and tail ends of the two zooids develop prior to the physical separation of the fusioning individual. This process is accompanied by remodeling of the original ventral nerve cord, development of the new brain, ventral ganglia, peripheral nerves, and sensory organs, including the eyes. Results of our study suggest that the involvement of *Pax6* in development and patterning of the nervous system is evolutionary conserved, despite the variability and evolution of the developmental trajectories.

## 1. Introduction

Pax6 is the most extensively studied among the transcription factors encoded by genes in the *Pax* family. *Pax6* homologs are important, evolutionarily conserved participants in the development of sensory structures, particularly the visual apparatus and neuroectoderm elements [1]. Experiments with targeted ectopic expression of the mouse *Pax6* gene in *Drosophila*, which resulted in the formation of ectopic eyes in the fly [2], contributed to the concept of a “master control gene” in eye development and even in the Evo-Devo field as a whole [3,4].

Like other Pax proteins, Pax6 has a DNA-binding domain called a paired domain (PD), which was originally discovered in *Drosophila* [5]. However, like some other members of the Pax family, Pax6 possesses a homeodomain (HD), which is also important for DNA binding. The structure and function of Pax6 are well conserved in bilateral animals [1,6,7,8].

Functional studies suggest that both the paired domain and the homeodomain are required for function. Furthermore, they can interact cooperatively and independently with target DNA, thereby ensuring their differential expression [9,10]. However, the paired domain plays a critical role in many cases. For example, it plays a key role in brain development; mutants lacking the Pax6 homeodomain exhibit only minor defects [11].

The Pax6 transcription factor binds to sequence-specific DNA and positively or negatively regulates transcription. It is expressed in many cell types of the developing and adult central nervous system (CNS) and sensory organs. It is also involved in a variety of biological processes by regulating the expression of a wide range of molecules, including transcription factors, cell adhesion and signaling molecules, hormones, and structural proteins reviewed in [1,12]. However, the key role of Pax6 in eye and brain development in both vertebrates and invertebrates remains undeniable, making it an intriguing subject of evolutionary research.

The involvement of *Pax6* in lens formation in vertebrates is well-established. In particular, *Pax6* expression in the lens placode is essential for transcriptional activation of crystallin genes [13,14,15]. During newt lens regeneration, *Pax6* is critical not only for activating crystallin expression, but also for promoting cell proliferation. The activity of *Pax6* homologs has also been demonstrated during the development of sensory organs in the neural retina, corneal epithelium, and olfactory placodes [11,16,17,18]. In cave-blind fish (*Astyanax mexicanus*), degeneration of ocular structures, particularly the lens, is associated with reduced *Pax6* expression levels during embryonic development [19].

*Pax6* expression during eye formation has been described repeatedly in Ecdysozoa, most notably *Drosophila melanogaster*, as well as the onychophorans *Euperipatoides kanangrensis* and *E. rowelli*, the myriapod *Glomeris marginata*, the spider *Parasteatoda tepidariorum*, the mite *Archegozetes longisetosus*, and others [4,20,21,22,23,24,25,26].

In CNS development, expression of *Pax6* homologs is evident already at the initial stages and continues throughout the formation period, often even in adult animals. Thus, in vertebrates, *Pax6* is observed in the neural plate and neural tube. Its expression continues as various brain regions form. Mutations in this gene lead to various anomalies of the CNS and sensory organs [1,12,27,28,29].

In vertebrates, including humans, *Pax6* is expressed in neural stem cells during embryonic development, as well as during postnatal and adult neurogenesis. *Pax6* is involved in regulating cell division, cell migration, and interkinetic nuclear migration (INM) during cell proliferation in the cerebral cortex [1,30,31,32,33,34]. In *Drosophila*, *Pax6* homologs, *eyeless* and *twin of eyeless*, exhibit differential activity, yet they are also involved in neurogenesis at all stages, including neuronal cell migration [20,35].

*Pax6* regulates the activity of several proneural genes and participates in Notch signaling during neurogenesis. It maintains its activity in some differentiated cells of the nervous system and associated cells, such as astrocytes [1,34,36,37,38,39]. From the initial stage of the CNS development onward, *Pax6* plays a key role in regionalizing the neuroectoderm and neural tube. Later, it is involved in the dorsoventral patterning of the mammalian telencephalon [1,32,40,41,42]. Additionally, *Pax6* influences the regionalization of the forebrain and the establishment of boundaries between developing forebrain regions in chordates, including invertebrates [7,29,43].

In contrast to vertebrates and ecdysozoans, the expression and function of *Pax6* in lophotrochozoans are understudied. However, existing data suggest that *Pax6* homologs are expressed during nervous system and eye development [44,45,46,47,48], regeneration [49,50,51], and may exhibit atypical activity due to body plan innovations, as seen in cephalopods [52,53]. On the other hand, most studies investigating *Pax6* expression in lophotrochozoans have focused on the embryonic and larval stages, despite the diversity of postembryonic developmental trajectories in these animals.

Annelids are one of the main groups of lophotrochozoans. Despite the stereotypical nature of their embryonic development, they are distinguished by their diverse anatomies and habitats of adult forms. Many species exhibit indirect development, while others are direct-developers [54,55,56,57]. Interestingly, annelids differ in their ability to regenerate caudal and head structures, including the ventral nerve cord, brain, and sensory organs [57,58,59,60]. Furthermore, many annelid species are capable of asexual reproduction by transverse fission. During this process, entire sections of the central nervous system and sensory organs develop de novo [47,59,60,61].

Naidid annelids, tiny, transparent, freshwater segmented worms, are an excellent model for studying the evolution of developmental trajectories and mechanisms [47,61,62,63,64,65]. In laboratory culture, they reproduce asexually only. During this process, within a mid-body segment, the animals form a new posterior (tail) end and a new anterior (head) end with several head segments in the anterior and posterior zooids (individuals), respectively. This type of fission is called paratomy, and as a result, the original animal is temporarily transformed into a chain of two or more zooids. After forming the new head and tail ends, the zooids physically separate from each other [47,61,62,63,65].

In this study, we identified *Pax6* homologs in the annelid *Nais communis* for the first time and demonstrated their expression during regular growth and asexual reproduction. Using in situ hybridization and immunohistochemical labeling of the nervous system, we discovered that the duplicated *Pax6* homologs are activated during the development of new nervous system and sensory elements, exhibiting different expression patterns. Thus, our study suggests that the involvement of *Pax6* in development and specification of the nervous system is evolutionary conserved, despite the diversity of the developmental trajectories. Differences in the expression patterns of paralogs may indicate that these genes have diversified their functions.

## 2. Materials and Methods

### 2.1. Animal Material and Fixation

A laboratory culture of *Nais communis* was maintained in Petri dishes with artificial spring water and Chlorophyta algae at 18 °C. As described previously [47,65], the animals were fed mashed spinach or dried spirulina powder; to optimize the intensity of reproduction in the cultures, artificial lighting was used (16 h day, 8 h night). In order to synchronize and stimulate the animals to undergo paratomic fission, worms from the main laboratory culture were transferred in separate Petri dishes with clean water, starved for a week, and then fed. Over the next nine days, animals were fixed daily in batches to obtain all stages of fission as well as growing adults. A day before fixations, the worms were placed overnight in Petri dishes with clean water to remove any food particles from their intestines. Prior to fixations, animals were relaxed for 10 min in relaxant solution (10 mM MgCl_2_/5 mM NaCl/1 mM KCl/8% ethanol; see [47,61,62]. Specimens were fixed overnight in 4% formaldehyde in 0.75 × PBS/0.1% Tween-20 (PTw) at 4 °C. For in situ hybridization experiments, the specimens were then stored in MeOH at −20 °C; for immunohistochemistry, the fixed specimens were transferred through a graded ethanol series and stored in 70% ethanol at −20 °C.

### 2.2. Sequence Retrieval, Gene Cloning, and Phylogenetic Analysis

The sequences of *Pax6* homologs were identified in an unannotated *N. communis* transcriptome database (local resource) [47]. Fragments for *Nco-pax6A*, *Nco-pax6B*, *Nco-pax6C*, and *Nco-pax6D* genes were amplified by routine and RACE (5′-RACE and 3′-RACE) PCR with gene-specific primers (Supplementary Materials, Table S1) and mixed-stage fission cDNA prepared with a SMARTer RACE cDNA amplification kit (Clontech, Cat. #634923, Mountain View, CA, USA). The amplified gene fragments were inserted into pCRII vectors (Invitrogen, Cat. #K4600-01, Invitrogen, Waltham, MA, USA). The obtained vectors with gene fragment were then used in the transformation of chemically competent *E. coli* (One Shot^®^ TOP10; Invitrogen, Cat. #K4600-01). Plasmids with correct inserts were checked by sequencing and used for synthesis of the digoxigenin-labeled RNA probes. Phylogenetic analysis was used to confirm the identity of cloned gene fragments (see below). As a result, the obtained fragments for *Nco-pax6A* and *Nco-pax6D* include complete CDS, and 5′ and 3′ UTR, whereas *Nco-pax6B* and *Nco-pax6C* include incomplete CDS (however, complete paired domain and homeodomain) and 3′ UTR. The sequences of *Nco-pax6A* (1860 bp), *Nco-pax6B* (1835 bp), *Nco-pax6C* (1842 bp), and *Nco-pax6D* (2003 bp) were deposited in GenBank with the accession numbers PX514181-PX514184.

For phylogenetic analysis, the homologous proteins of interest were searched in the NCBI protein database. To establish homology, we employed BLASTp. The web service PROSITE was used to establish the domain organization of the sequences (https://prosite.expasy.org/, accessed on 15 October 2025). Amino acid alignment of the fragments contained both paired domain and homeodomain was performed with MAFFT [66,67] using online tools (https://www.ebi.ac.uk/jdispatcher/msa/mafft, accessed on 15 October 2025). The result of multiple alignment (FASTA format) is given in the Supplementary Materials, Tex S1. Bayesian phylogenetic analysis was conducted using the MrBayes 3.2.7_0 online tools (https://ngphylogeny.fr, accessed on 15 October 2025) [68,69]; the GTR model was chosen, while rate variation across sites was fixed to “equal”. Four Markov Chain Monte Carlo (MCMC) chains were run for 100,000 generations, sampling every 500 generations, with the first 250 sampled trees discarded as “burn-in”. Finally, a 50% majority rule consensus tree was constructed. The phylogenetic tree was and visualized by iTOL tools (https://ngphylogeny.fr, accessed on 15 October 2025) [70] (Supplementary Materials, Figure S1).

### 2.3. Immunohistochemistry

To describe in more detail the nervous system architecture of *N. communis*, immunohistochemistry against acetylated-α-tubulin was carried out according to the previously published protocol [47,55], with some modifications. The growing adults and asexual reproducing animals were first relaxed using anesthesia and then fixed as described above (Section 2.1. Animal material and fixation). Unless stated otherwise, all steps of the immunohistochemical studies were carried out at room temperature. After storage in 70% ethanol, specimens were rehydrated stepwise to PTw. Then they were permeabilized with 0.1% Triton-X in PBS (PBT), blocked 1 h in 5% normal sheep serum (Sigma, Cat. #S2263, Burlington, MA, USA) in PBT, and incubated with anti-acetylated-α-tubulin monoclonal antibody (T6793, Sigma, St. Louis, MO, USA) diluted 1:250 in PBT with 2.5% normal sheep serum, overnight at 4 °C. Specimens were then washed in PBT several times and incubated in PBT with Alexa-Fluor-488-conjugated anti-mouse antibody (diluted 1:400; Invitrogen, Waltham, MA, USA, Cat. # A-11001) for 2 h at room temperature. After washing with PTw, specimens were transferred through a graded glycerol series, mounted in 90% glycerol in PBS and examined by confocal laser scanning microscopy.

### 2.4. Whole-Mount In Situ Hybridization

The whole-mount in situ hybridization (WMISH) experiments were carried out according to the previously published protocol [47] with several modifications. As the templates for the digoxigenin-labeled RNA probes (both antisense and sense) the plasmids with inserted gene fragments of about 1290 bp (*Nco-pax6A*), 1423 bp (*Nco-pax6B*), 1217 bp (*Nco-pax6C*), and 1170 bp (*Nco-pax6D*) were used. Specimens were transferred from MeOH to PTw, rinsed several times in PTw, permeabilized with Proteinase K (100 µg/mL; Merck, Cat. #1.24568.0100, Sigma-Aldrich Chemie GmbH, Taufkirchen, Germany) for 2 min at +22 °C, washed twice in glycine (2 mg/mL), and then postfixed in 4% PFA for 20 min. The samples were washed several times in PTw prior to the pre-hybridization step. Incubation with the DIG-probe was performed at +65 °C. The specimens were then washed and incubated with anti-digoxigenin AP antibodies (1:2500; Roche, Cat. #1093274910, Sigma-Aldrich Chemie GmbH, Taufkirchen, Germany). After washing, the samples were stained with NBT/BCIP (Roche, Cat. #11383213001/11383221001, Sigma-Aldrich Chemie GmbH, Taufkirchen, Germany). After washing in PTw, they were mounted in 90% glycerol. Three independent sets of in situ hybridization experiments were performed. In each set of hybridizations with an antisense RNA probe, we used 20 samples for each stage of asexual reproduction or growing worms. In situ hybridization with the sense DIG-labeled riboprobe was used as a negative control (Supplementary Materials, Figure S2).

### 2.5. Data Visualization

After immunohistochemistry, specimens were imaged using a Leica SP5X confocal laser scanning microscope (Leica, Wetzlar, Germany) under 40× oil immersion or 60× glycerol lenses. An argon laser with a wavelength of 488 nm was used to detect AlexaFluor 488. Z-stacks with 1.0 mkm steps were acquired using the Leica LAS X Office software. A maximum projection of a confocal Z-stack was used as a technique to create a single 2D image. After in situ hybridization, imaging of the mounted glycerol specimens was conducted using DIC optics on an Axio Imager D1 microscope (Carl Zeiss, Oberkochen, Germany). Digital photomicrographs were taken with an AxioCam ICc3 digital camera using the AxioVision 4.8 software (Carl Zeiss, Oberkochen, Germany). The artworks were made in ImageJ (version 1.51), MS PowerPoint (Microsoft Office 2013), and Adobe Photoshop CS5.

## 3. Results

**Figure 1:**
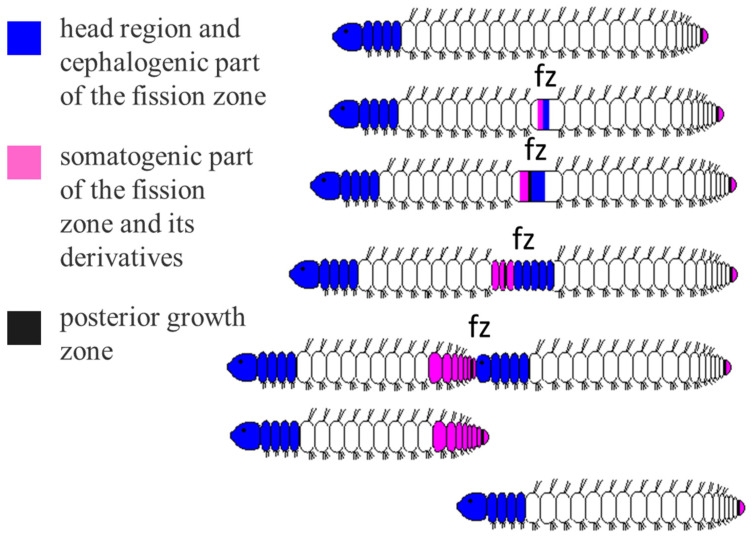
Schema of paratomic fission in *Nais communis*. A fission zone (fz) is typically formed between segments 12 and 23, within a mid-body segment. It gives rise to a new cephalic region (head and four head segments), and a new tail end with pygidium, growth zone, and some trunk segments. The paratomy process is continued by forming transiently linked chains of individuals (zooids) which then split apart once development of new body regions is complete.

In long-term laboratory culture, *Nais communis* reproduce only by paratomy, a type of asexual propagation in which new head and tail ends develop within a mid-body segment before the newly formed individuals physically separate. Ready to undergo asexual reproduction *N. communis* worms are typically comprising 21–41 segments [47,65]. At the anterior end of the worm, there is a head (cephalic region) consisting of the prostomium, peristomium, and four specialized head (cephalic) segments. At the posterior end, just anterior to the pygidium, the posterior growth zone is located; it produces new segments during normal growth. There is no fixed segment for developing the paratomic zone: a fission zone is usually formed between segments 12 and 23. Paratomic fission of naidids is well morphologically described [61,62,65]. Development of the fission zone is characterized by formation of blastema masses of undifferentiated (possibly dedifferentiated) cells, active cell proliferation, and remodeling of old tissues. At the beginning, the fission zone is morphologically indistinguishable; however, very soon it forms two regions with a distinct border (Figure 1). The anterior part of the fission zone called somatogenic develops a new posterior end of the anterior zooid. The posterior part (cephalogenic) of the fission zones gives rise to a new head end (including four head segments) of the posterior zooid [47,65]. The physical separation of the two daughter individuals occurs once development of new body regions is complete.

In this work, we identified in *N. communis* four homologs of *Pax6* (*Nco-pax6A*, *Nco-pax6B*, *Nco-pax6C*, and *Nco-pax6D*) and examined their developmental patterns by WMISH. To gain better understanding expression patterns of these paralogs, we used immunohistochemistry and confocal laser scanning microscopy to describe the nervous system architecture of the growing and asexually reproducing *N. communis* worms.

### 3.1. Architecture of the Nervous System of a Growing Worm

**Figure 2:**
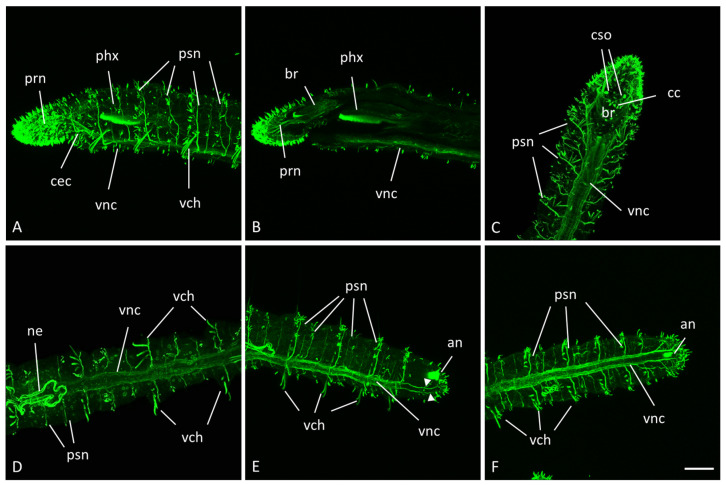
Nervous system architecture of the growing adult *N. communis* worms. Immunohistochemistry against acetylated-α-tubulin. Maximum projections of confocal Z-stacks scanned from lateral side (**A**,**B**,**E**) or ventral side (**C**,**E**,**F**). Except for (**C**), all animals are oriented anterior to the left, in (**C**) anterior is up. (**A**–**C**), anterior end ((**B**), deep focal plane). (**D**)—a mid-body segment. (**E**,**F**)—posterior end. an—anus, br—brain, cec—circumesophageal connectives, cc—cerebral commissure, cso—ciliary sense organ, ne—nephridium, phx—pharynx, prn—prostomial nerve, psn—peripheral segmental nerve, vch—ventral chaetae (autofluorescence), vnc—ventral nerve cord. Peripheral nerves that innervate the pygidium, including the sensory hairs on the surface, are indicated by the arrowheads. Scale bar, 50 µm for all panels.

The nervous system of *N. communis* consists of three main components: the anterior brain, the ganglionated ventral nerve cord, and peripheral nervous system (Figure 2).

Anti-acetylated α-tubulin antibodies label nerve fibers of the central and peripheral nervous systems. However, they also detect other structures, such as the cilia of cells in the digestive tract and nephridia.

The brain, the anterior part of the central nervous system, is a paired bilobed structure located dorsal to the mouth. The right and left lobes are connected by the cerebral commissure (Figure 2C). The brain is linked to the ventral nerve cord by paired circumpharyngeal connectives (Figure 2A,B). The circumpharyngeal connectives are also connected to paired groups of prostomial nerves of the peripheral system, innervating the prostomium and the dense cluster of epidermal sensory hair cells on its surface (Figure 2A–C). Near the posterior edge of the prostomium, a pair of lateral pigmented cup-shaped eyes are located.

The ventral nerve cord, another part of the central nervous system, runs longitudinally along the entire length of the animal (Figure 2). Each segment contains one ganglion. At the anterior end of the cord, there is an additional ganglion, called the subesophageal ganglion. Laterally, segmental nerves of the peripheral system branch off from the ventral nerve cord and run to the dorsal side of the worm. Each nerve innervates a set of epidermal sensory hairs. No segmental nerves are observed at the posterior end of the worm. Instead, peripheral nerves arising from the ventral nerve cord branch and innervate the pygidium, including the sensory hairs on the surface (Figure 2E,F).

### 3.2. Development and Remodeling of the Nervous System Architecture During Asexual Reproduction

**Figure 3:**
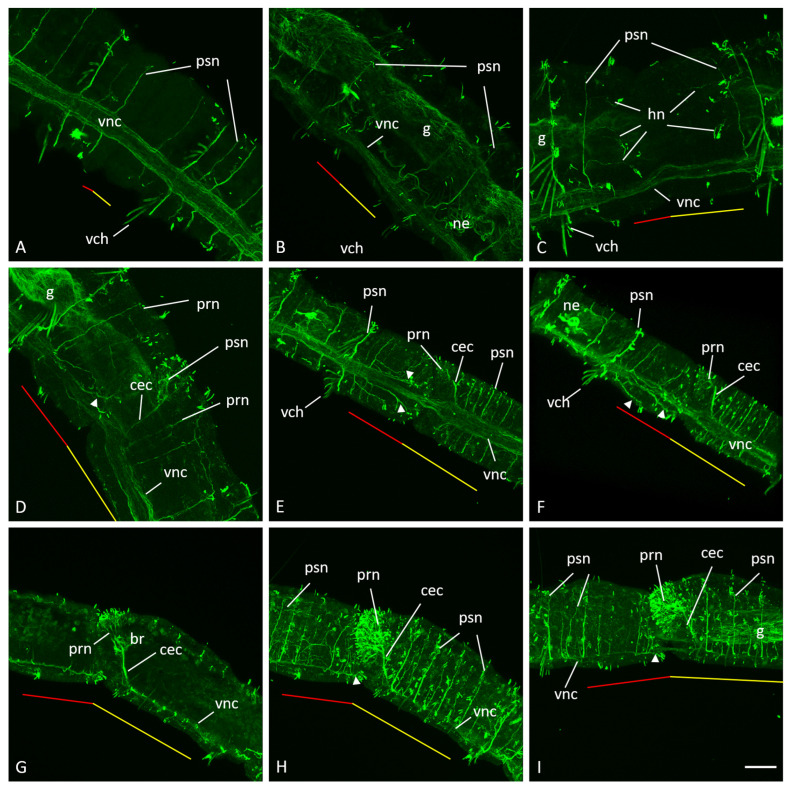
Remodeling of nervous system architecture during asexual reproduction in *N. communis*. Immunohistochemistry against acetylated-α-tubulin. Maximum projections of confocal Z-stacks scanned from ventral side (**A**,**E**) or lateral side (**B**–**D**,**F**–**I**). Animals are oriented anterior to the upper left corner (**A**,**B**,**D**–**F**) or anterior to the left (**C**,**G**–**I**). (**A**,**B**)—Early fission stage. (**C**,**D**)—Mid fission stage. (**E**–**I**)—Late fission stage ((**G**), deep focal plane). br—brain, cec—circumesophageal connectives, cc—cerebral commissure, g—gut, hn—horizontal nerve, ne—nephridium, prn—prostomial nerve, psn—peripheral segmental nerve, vch—ventral chaetae (autofluorescence), vnc—ventral nerve cord. The red line marks the new developing tail region, the yellow line marks the new developing head region within the fission zone. The arrowheads indicate peripheral nerves that innervate the new posterior end. Scale bar in (**A**–**D**), 25 µm; in (**E**–**I**), 50 µm.

At the initial steps of asexual reproduction, no changes in the nervous system architecture are observed within the segment forming the paratomy zone (Figure 3A). As the blastemal cell mass forms and grows, a localized narrowing of the ventral nerve cord becomes visible at the boundary of the cephalogenic and somatogenic parts of the fission zone (Figure 3B). During the growth of the blastemal mass at the early fission stage, the paratomy zone lacks segmental nerves (Figure 3A–C).

At the middle stage of asexual reproduction, growth of horizontal nerves innervating the blastemal masses is observed (Figure 3C). These nerves branch off from the old segmental nerves of the peripheral nervous system. From the nerve located in front of the blastemal masses, they run posteriorly, and from the nerve located behind the blastemal masses, they extend anteriorly (Figure 3C). The paratomy zone increases in length. Soon later, at the middle stage of development, the architecture of the nervous system in the somatogenic and cephalogenic parts shows significant differences (Figure 3D). The original ventral nerve cord of the organism retains its architecture, but it is narrowed at the border of the somatogenic and cephalogenic parts. In the cephalogenic part, the ventral nerve cord becomes wider than in the somatogenic part. Segmental nerves and circumesophageal connectives branch off from it (Figure 3D). The circumesophageal connectives begin to grow in an anterior direction dorsally from the ganglion of the cephalogenic part of the paratomy zone closest to the border of the posterior zooids. Nerves then branch off from them, innervating the prostomium. Sensory hairs become visible on the surface of the prostomium (Figure 3D). The new posterior end of the ventral nerve cord forms more ventrolaterally than the original nerve cord (Figure 3D). The nerves formed from the segmental nerves continue to elongate. They are especially noticeable in the somatogenic part (Figure 3D). These nerves innervate the sensory hairs.

At the late stage of the fission, all the main components of the nervous system of the new tail end of the anterior zooid and the new cephalic region of the posterior zooid are formed (Figure 3E–I). The cerebral commissure appears, connecting the right and left lobes of the brain. New segmental nerves form in the new segments. In the somatogenic part, peripheral nerves growing from the posterior end of the ventral nerve cord branch and surround the future pygidium (Figure 3). The old nerve cord remains continuous until the physical separation of two individuals.

### 3.3. Pax6 Homolog Expression in Growing Adults Nais communis

**Figure 4:**
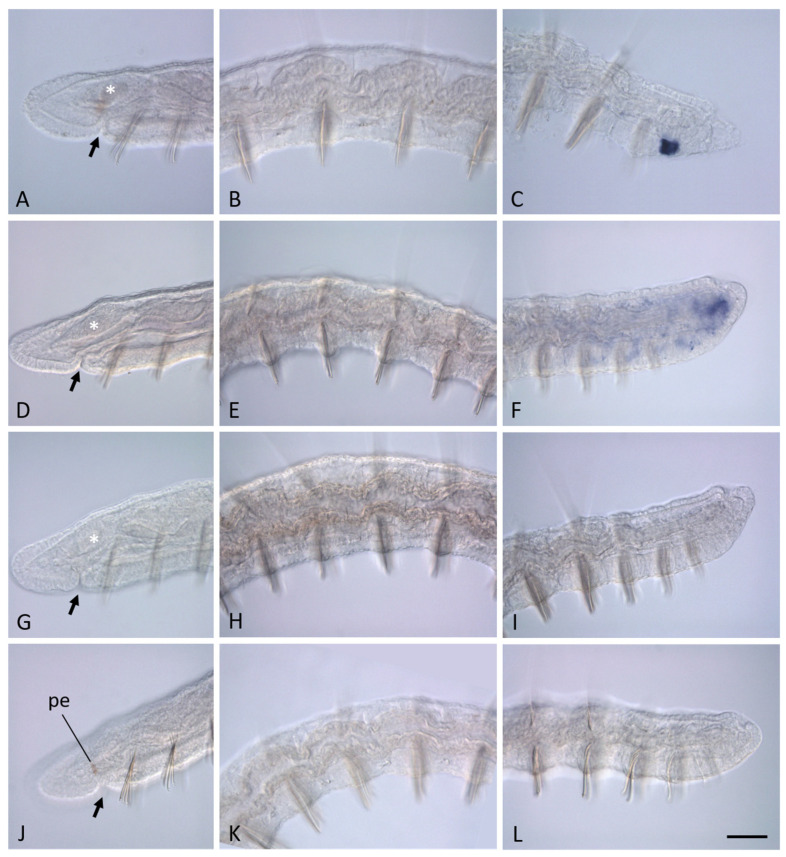
*Pax6* homolog expression in growing adults *N. communis*. All animals are oriented anterior to the left. Lateral view for all panels. (**A**–**C**), *Nco-pax6A* expression, (**D**–**F**), *Nco-pax6B* expression, (**G**–**I**), *Nco-pax6C* expression, (**J**–**L**), *Nco-pax6D* expression. Anterior end of the worms (**A**,**D**,**G**,**J**); mid-body region (**B**,**E**,**H**,**K**); posterior end (**C**,**F**,**I**,**K**). None of the *Pax6* homologs show its activity in the head region and mid-trunk segments. (**C**) *Nco-pax6A* is strongly expressed at the posterior end of the worms (just anterior to the posterior growth zone. *Nco-pax6B* signal is more diffuse and observed in the deep cells of the posterior growth zone, as well as in some cells of the ventral nerve cord of the posterior segments of the worm. pe—pigmented eye. Mouth is indicated by arrow; the asterisk marks the brain. Scale bar, 40 µm for all panels.

In non-fissioning growing adults *N. communis*, transcripts of none of the *Pax6* paralogs were detected in the head region and mid-trunk segments by in situ hybridization (Figure 4). Furthermore, *Nco-pax6C* and *Nco-pax6D* expression was not observed at the posterior end, including young segments, the posterior growth zone and pygidium (Figure 4). *Nco-pax6A* is strongly expressed in the terminal region of the ventral nerve cord of the youngest segments located anterior to the growth zone (Figure 4C). Transcripts of this gene were not detected in the cells of the growth zone and pygidium. In contrast, *Nco-pax6B* expression was shown in deep cells of the growth zone (Figure 4F). Furthermore, low levels of transcripts were detected in some cells of the ventral nerve cord of the posterior segments of the animal. Like other paralogs, *Nco-pax6B* does not show its activity in the pygidium.

Interestingly, starved adult animals stop growing [61,63]. In such animals, no signs of expression of either homolog were detected by in situ hybridization. After feeding, the animals begin to grow again and show signs of expression. This phenomenon was previously reported for other naidid species, *Prisitina leidyi* [63].

### 3.4. Expression of Pax6 Homologs in Asexually Reproducing Nais communis

#### 3.4.1. Expression of *Pax6* Homologs During the Early-Fission-Stage

**Figure 5:**
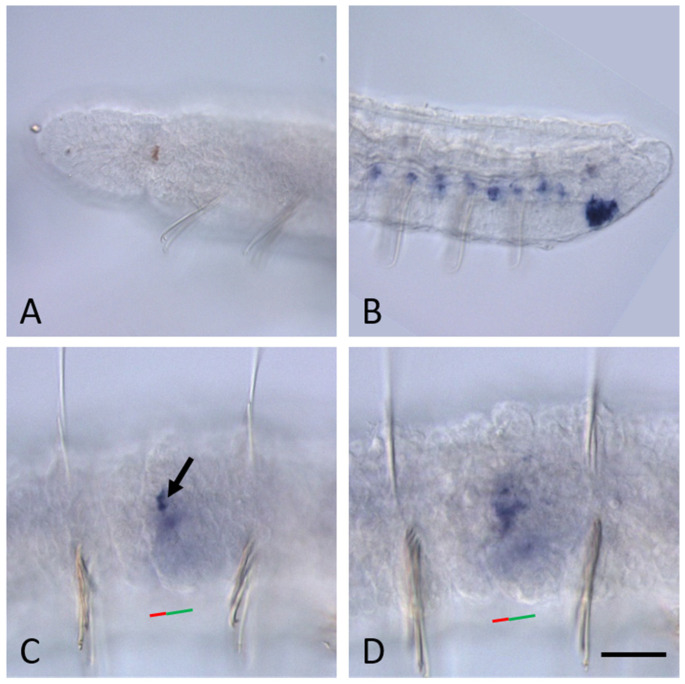
*Nco-pax6A* expression during asexual reproduction in *N. communis*. Early fission stage. Animal is oriented anterior to the left. Lateral view for all panels. Anterior end of the fissioning worm (**A**); posterior end of the same animal (**B**); mid-body segment, where *Nco-pax6A* appears to be expressed de novo in the developing fission zone (**C**)—strong expression in the superficial cells is indicated by arrow, ((**D**)—expression in the deep cells, deep focal plane). The red line marks the new developing tail region, the green line marks the new developing head region within the fission zone. Scale bar in (**A**,**B**), 40 µm; in (**C**,**D**), 25 µm.

During the earliest steps of asexual reproduction, when the integumentary epithelium becomes modified within one of the mid-trunk segments [47,65], none of the *Nco-pax6* genes are expressed in the developing paratomy zone. Soon, at the onset of blastema mass formation, *Nco-pax6A* is de novo expressed in several patches of cells domains within the fission zone (Figure 5). Strong expression is observed in two small bilateral domains located laterally and consisting of a few integumentary epithelial cells (Figure 5C). High levels of *Nco-pax6A* transcripts are also detected in two bilateral internal domains within the blastema masses (Figure 5D). At the end of the early fission stage, when the boundary between the cephalogenic and somatogenic parts appears (the epidermal groove begins to form), it becomes apparent that *Nco-pax6A* expression domains are observed in the newly developing head region. Within the developing paratomy zone, mRNA of the other three genes (*Nco-pax6B*, *Nco-pax6C*, and *Nco-pax6D*) is not detected by in situ hybridization at the early-fission stage. The expression pattern of these genes at the anterior and posterior ends of the parental individual remains unchanged during this stage. No signs of *Nco-pax6A* expression in the head region of the anterior zooid is observed. In contrast, in the caudal end of the posterior zooid, *Nco-pax6A* transcripts are shown not only in the terminal region of the ventral nerve cord of the youngest segments but also in individual cells localized dorsolaterally in the ganglia of the ventral nerve cord of developed segments (Figure 5B).

#### 3.4.2. Expression of *Pax6* Homologs During the Mid-Fission-Stage

**Figure 6:**
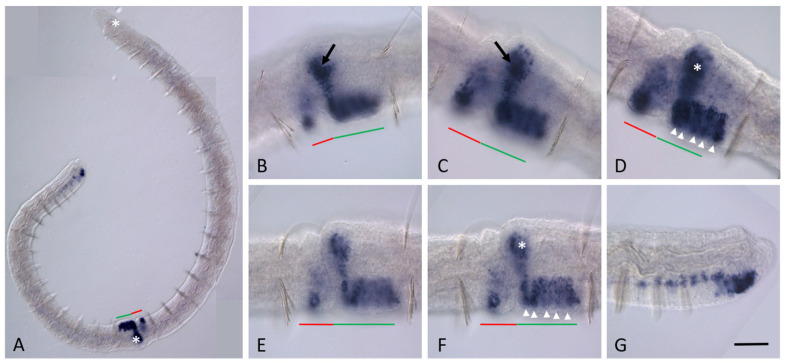
*Nco-pax6A* expression during asexual reproduction in *N. communis*. Lateral view for all panels. An asexually reproducing worm at the middle stage of the paratomy zone development (**A**); anterior is up. *Nco-pax6A* expression within the paratomy zone at the mid-fission stage (**B**–**D**) and the late-fission stage (**E**,**F**), and at the tail end of the posterior zooid at the late-fission stage (**G**). Animals is oriented anterior to the left. (**C**,**D**) show the same specimen; (**D**), deep focal plane. (**E**,**F**) show the same specimen; (**F**), deep focal plane. Expression in the superficial cells is indicated by arrow; the asterisk marks the brain anlage; the new developing ganglia of the ventral nerve cord within the new head region are indicated by arrowheads. The red line marks the new developing tail region, the green line marks the new developing head region within the fission zone. Scale bar in (**A**), 125 µm; in (**B**–**G**), 40 µm.

At the mid-fission stage, expression of all four *Pax6* homologs (*Nco-pax6A*, *Nco-pax6B*, *Nco-pax6C*, *Nco-pax6D*) is detected within the fission zone. Particularly high transcript levels are observed for *Nco-pax6A* (Figure 6A–D). In the newly developing head region, this gene shows strong expression in broad bilateral domains of superficial and deep cells located dorsolaterally at the anterior part of the cephalogenic region, as well as in the circumpharyngeal region. In addition, *Nco-pax6A* shows its activity in the internal cells of the ventral and ventrolateral domains of the developing head region. At this stage of the paratomy zone development, *Nco-pax6A* is de novo expressed in the superficial cells as well as in the internal cells of the ventral and ventrolateral domains of the developing tail. Thus, within the fission zone, the expression domains of this gene correspond to the localization of cells that give rise to the brain, the suboesophageal ganglion and ganglia of the ventral nerve cord, the dense cluster of epidermal sensory hair cells on the surface of the prostomium and pygidial region, and the eyes.

**Figure 7:**
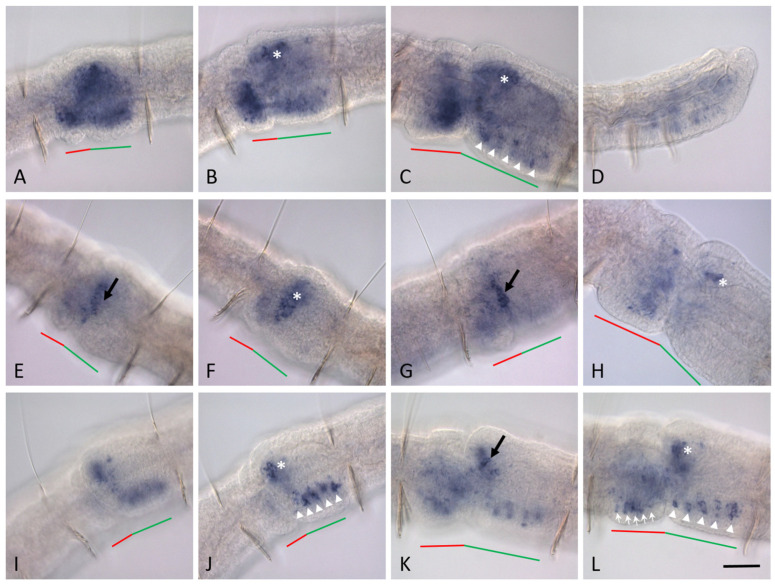
*Pax6* homolog expression during asexual reproduction in *N. communis*. All animals are oriented anterior to the left. Lateral view for all panels. *Nco-pax6B* expression within the paratomy zone at the mid-fission stage (**A**,**B**) and the late-fission stage (**C**), and at the tail end of the posterior zooid at the late-fission stage (**D**). *Nco-pax6C* expression within the paratomy zone at the mid-fission stage (**E**–**G**) and the late-fission stage (**H**). (**E**,**F**) show the same specimen; (**F**), deep focal plane. *Nco-pax6D* expression within the paratomy zone at the mid-fission stage (**I**,**J**) and the late-fission stage (**K**,**L**). (**I**,**J**) show the same specimen; (**J**), deep focal plane. (**K**,**L**) show the same specimen; (**L**), deep focal plane. Expression in the superficial cells is indicated by black arrow; the asterisk marks the brain anlage; the new developing ganglia of the ventral nerve cord within the new head region are indicated by arrowheads; the white arrows mark ganglia of the ventral nerve cord within the new tail region. The red line marks the new developing tail region, the green line marks the new developing head region within the fission zone. Scale bar, 40 µm for all panels.

During the middle stage of development, *Nco-pax6B*, *Nco-pax6C*, and *Nco-pax6D* are expressed de novo at the developing head end (Figure 7). All of them exhibit a more diffuse pattern than *Nco-pax6A*, but their expression patterns differ in detail. Thus, *Nco-pax6B* transcripts are observed in a broad region corresponding to internal masses of undifferentiated cells (Figure 7A,B). In addition, activity of this gene is detected in several superficial cells located dorsolaterally on both sides of the developing head region. *Nco-pax6C* expression is shown in two narrow bands of superficial cells adjacent to the epidermal fold and located on both sides of the cephalogenic part (Figure 7E,G). Additional internal domains of expression of this gene are also detected in the anterior part of the developing head end. These domains correspond to cells of the blastemal masses located dorsally and laterally (Figure 7F). *Nco-pax6D* transcripts are detected in a few anterior dorsolateral cells in the cephalogenic part, but the major expression domains of this gene are observed within the developing brain and ventral nerve cord (Figure 7I,J).

During the mid-fission stage, *Nco-pax6B* is highly expressed in the developing tail end, while *Nco-pax6C* transcript levels are low and *Nco-pax6D* expression is very weak (Figure 7A,B,E–G,I,J).

Interestingly, by the end of the mid-fission stage, expression of three genes (*Nco-pax6A*, *Nco-pax6B*, and *Nco-pax6D*) shows a metameric pattern in ventral domains of the developing head region (Figure 6D and Figure 7J).

#### 3.4.3. Expression of *Pax6* Homologs During the Late-Fission-Stage

**Figure 8:**
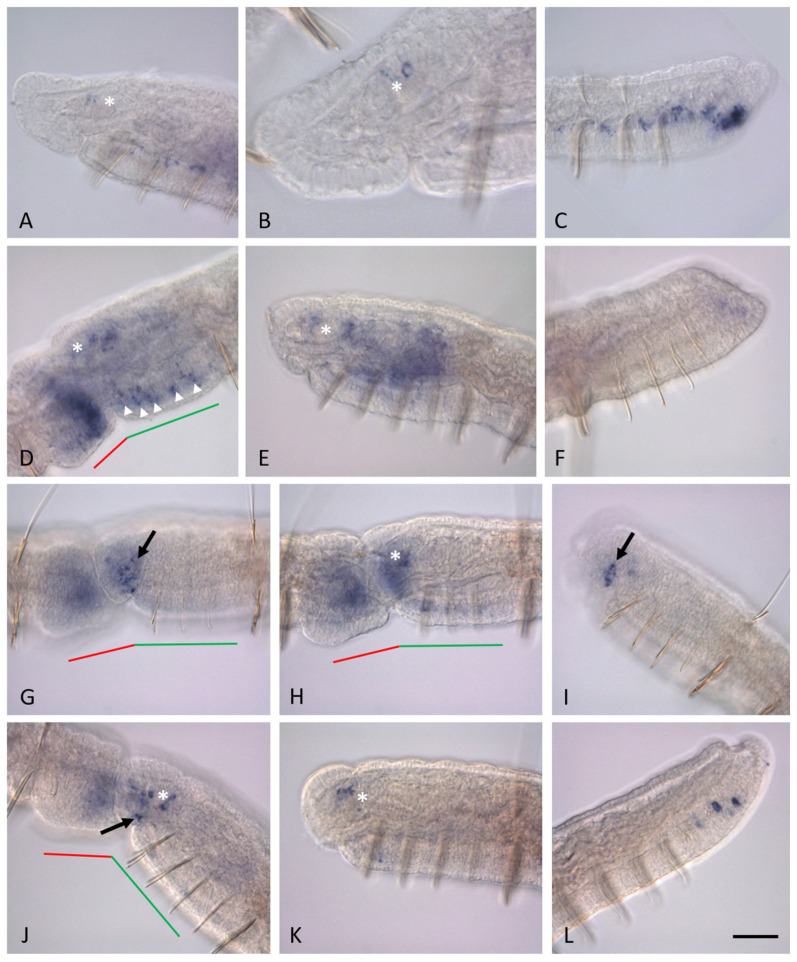
*Pax6* homolog expression in asexually reproducing *N. communis*. All animals are oriented anterior to the left. Lateral view for all panels. *Nco-pax6A* expression in the new head region of the posterior zooid (**A**,**B**) and new tail end of the anterior zooid (**C**) after physical separation of new individuals from each other. *Nco-pax6B* expression before physical separation of new individuals from each other ((**D**), fission zone) and immediately after physical separation (**E**), new head region of the posterior zooid; ((**F**), new tail end of the anterior zooid). *Nco-pax6C* expression before physical separation of new individuals from each other ((**G**,**H**), fission zone; (**H**), deep focal plane) and immediately after physical separation ((**I**), new head region of the posterior zooid). *Nco-pax6D* expression before physical separation of new individuals from each other ((**J**), fission zone) and immediately after physical separation (**K**), new head region of the posterior zooid; ((**L**), new tail end of the anterior zooid). Expression in the superficial cells is indicated by black arrow; the asterisk marks the brain anlage; the new developing ganglia of the ventral nerve cord within the new head region are indicated by arrowheads. The red line marks the new developing tail region, the green line marks the new developing head region within the fission zone. Scale bar, 40 µm for all panels except (**B**). Scale bar in (**B**), 20 µm.

*Nco-pax6A* expression levels remain very high throughout the mid-fission stage in both the developing tail end and the developing head end. However, during the late fission stage, expression weakens. Animals at this stage possess visible boundaries between the anterior and posterior zooids, developed eyes; segmentation in the fission zone becomes morphologically evident. At the beginning of the late stage, in the ventral region of the cephalogenic part of the paratomy zone, the *Nco-pax6A* expression pattern retains a metameric character (Figure 6E,F). Later, *Nco-pax6A* expression gradually disappears and is limited to only low levels of transcripts in some cells of the central nervous system. A few cells located dorsally within the developing brain, as well as some dorsal cells of the ventral nerve cord ganglia of the new head segments, show a weak in situ signal even after physical separation of the posterior zooid (Figure 8A,B). However, very soon this expression completely disappears. The new caudal end of the anterior zooid, as well as the old caudal end of the posterior zooid, rapidly develops new segments due to the activity of the growth zone [47,65]. In both cases of active growth of individuals, *Nco-pax6A* expression was observed not only in the terminal region of the ventral nerve cord of the youngest segments, but also in some dorsolaterally localized cells of the ventral nerve cord of developed segments (Figure 6G and Figure 8C).

Throughout the late fission stage, diffuse *Nco-pax6B* expression is found in both the developing new caudal end and the developing head end (Figure 8D). In the cephalogenic part, the highest levels of *Nco-pax6B* mRNA are detected in the ganglion cells of the ventral nerve cord and in a few cells of the developing brain. Immediately after physical separation of the posterior zooid, expression is no longer observed in the ventral ganglion cells of the new head segments, while it persists for some time in a few anterior-dorsal and posterior cells of the brain (Figure 8E) before completely disappearing. Interestingly, diffuse expression of this gene in the internal tissues of the new head segments persists longer than in the ventral ganglion cells (Figure 8E). However, it soon disappears. The level of *Nco-pax6B* transcripts in the new caudal end decreases significantly by the time the new individuals separate (Figure 8F). At the late fission stage, *Nco-pax6C* expression is shown in superficial cells corresponding to epidermal sensory hair cells on the surface of the prostomium, as well as in the eye region (Figure 8G,H). In addition, *Nco-pax6C* transcripts are detected in some cells of the developing brain, the suboesophageal ganglion, and in the internal cells of the new caudal end. Immediately after physical separation of individuals from each other, expression domains are no longer detectable, with the exception of eye cells (Figure 8I). However, high levels of *Nco-pax6C* transcripts in the eyes are observed for a short time, as expression soon completely disappears.

The metameric pattern of *Nco-pax6D* expression in the developing ventral ganglia is observed throughout the late fission stage (Figure 7K,L). However, by the end of this stage, transcript levels decrease significantly. Before the physical separation of the zooids, mRNA for this gene is found in the anterior and posterior cells of the new brain and the new subesophageal ganglion, in several superficial cells surrounding the eyes, and in the region of the developing posterior segments (Figure 8J). Immediately after the physical separation of the zooids, expression begins to disappear, persisting for some time in the anterior dorsal cells of the new brain of the posterior individual and in some dorsolateral cells of the ventral ganglia of the young segments of the anterior individual (Figure 8K,L).

The expression pattern of all four genes at the anterior and posterior ends of the parental individual remains unchanged during the middle and late stages of fission zone development.

## 4. Discussion

*Pax6* plays a highly conserved role in the formation of the eye and nervous system in animal development [1,12]. Its expression patterns have been described in embryos and adults of many species. Data on the possible role of *Pax6* during regeneration are more limited and primarily relate to studies of eye, neural retina, and lens repair [15,51,71,72]. Despite the fact that asexual reproduction is widespread among metazoans, there are no detailed studies on *Pax6* involvement in such developmental trajectory as agametic propagation. The present study represents the first investigation of *Pax6* gene expression during asexual reproduction in annelids. To better understand the evolutionarily conserved role for *Pax6*, we identified homologs of this gene in the annelid *Nais communis*, demonstrated their expression, and characterized changes in nervous system architecture during regular growth and asexual reproduction.

### 4.1. Nervous System Architecture in Growing N. communis, Its Remodeling and Development During Asexual Reproduction

The nervous system architecture of the species we studied corresponds to the basic plan of clitellates [73] and consists of three main components: the brain, the ganglionic ventral nerve cord, and peripheral nerves connected to both parts of the central nervous system. The brain is linked to the ventral nerve cord by paired circumpharyngeal connectives. The prostomial nerves of the peripheral system innervate the prostomium and numerous epidermal cells of sensory hairs on its surface. In each segment, segmental nerves of the peripheral system extend from the ventral nerve cord. Peripheral nerves extend from the ventral nerve cord at the posterior end branch and innervate the pygidium, including sensory hairs on the surface.

During asexual reproduction, nervous system remodeling begins with the formation and growth of the blastemal cell mass in the developing fission zone. Initially, some localized narrowing of the ventral nerve cord is observed. Similar to regeneration, where the transected ventral cord plays a key role in initiating regeneration in annelids [58,60,62], this event likely results in some localized functional limitations in the paratomy zone and facilitates further growth of the blastemal mass and nervous system development.

At mid-fission, the appearance of numerous horizontal nerves that extend from nearby parental segmental nerves of the peripheral nervous system and innervate the blastemal masses has previously been described as a specific event of regeneration, but not of embryogenesis or growth, in oligochaetes [58,74,75]. However, such temporary peripheral nerves have also been demonstrated in paratomy of naidid annelids, *Pristina leidyi* and *N. communis* ([62]; this study). This similarity in the initial stages of nervous system remodeling may provide further evidence for the origin of paratomy based on regenerative capacities [59,60,62].

Within the developing fission zone, new ganglia of the central nervous system, the cerebral ganglion and the ganglia of the ventral nerve cord, are formed from the dorsal and ventral cell patches, respectively [61,62,65]. The old ventral nerve cord remains continuous until the physical separation of individuals. It gives rise to new nerve tracts. Interestingly, development of new nerve tracts in *N. communis* and representatives of the genus *Pristina* (*P. leidyi* and *P. longiseta*) demonstrate significant differences [47,62]. In *Pristina*, the formation of dual ventral nerve cords is observed in the fission masses at the late stage of paratomy zone development. In contrast, in *N. communis*, the circumpharyngeal connectives begin to grow anterodorsally from the ganglion closest to the zooid border in the cephalogenic part of the paratomy zone, and the nerves innervating the new posterior end branch laterally, showing more ventrolateral localization than the original nerve cord.

Thus, during fission, along with remodeling of the original ventral nerve cord, development of the new brain, ventral ganglia, peripheral nerves, and sensory organs, including the eyes, is observed.

### 4.2. Evolutionarily Conserved Role of Pax6 in Eye and Nervous System Development

Expression studies and functional analysis have shown that *Pax6* is an important regulator of eye and nervous system development across the major bilaterian lineages: Lophotrochozoa, Ecdysozoa, and Deuterostomia [1,4,11,12,16,17,18,20,21,22,23,24,25,26,44,45,46,47,48,49,50,51,52,53].

Among lophotrochozoans, *Pax6* expression associated with both eye development or regeneration and the nervous system has been shown in annelids [44,45,46,47,48,49,50], mollusks [52,53,76,77], planarians [51], nemerteans [72], and chaetognaths [78], including adult animals. For example, in the annelid *Platynereis dumerilii*, *Pax6* transcripts were detected in larval eye cells. In metatrochophore larvae, this gene is expressed in dorsal cells located at the base of developing adult eyes, as well as in mechano- and chemosensory cells of the palps and the tips of the developing antennae, which may serve as confirmation that *Pax6* is involved in the formation of not only the visual organs but also other sensory elements [44]. Furthermore, at the trochophore stage, *Pax6* is expressed on the ventral side as two longitudinal stripes, participating in the mediolateral patterning of the developing ventral nerve cord [45]. During posterior regeneration in *P. dumerilii*, *Pax6* is expressed de novo in neuroectoderm cells within the regenerate [49]. In *Capitella teleta*, the appearance of *Pax6* expression coincides with the onset of nervous system development. Functional experiments using morpholino knockdown have shown that disruption of the paired domain structure leads to significant disturbances in the development of the nervous system and ocular structures in the development of this annelid [48].

Many animals, including humans, have a single copy of the *Pax6* gene, but duplications of this gene have been shown in many animal groups. Two homologs have been described in some arthropods [20,24,25,26], mollusks [76], leeches [50], flatworms [51,79,80], teleosts [81,82], the elephant shark, *Xenopus tropicalis*, and the *Anolis* lizard [83]. Three *Pax6* genes have been found in lampreys [84]. Duplicated genes are typically differentially expressed in the eyes and nervous system but may also exhibit variable expression in other tissues. Differential expression of duplicated *Pax6* homologs in the sensory pits during regeneration and in the asexual phase was recently described for the flatworm *Stenostomum brevipharyngium* [80]. Together with the results of functional experiments, the differential expression pattern suggests functional diversification of the duplicated genes [20,51,79,81,82,83]. In this study, we identified four *Pax6* homologs in the asexually reproducing annelid *N. communis*. In situ hybridization analysis of their expression revealed that only two of them, *Nco-Pax6A* and *Nco-Pax6B*, are active in growing adult worms (in young segments at the posterior end). However, all four genes are expressed de novo within the developing fission zone during asexual reproduction. *Nco-Pax6A* is expressed earlier than the other genes, at the early fission stage. Its expression domains are observed in the newly developing head region, both on the surface and within the internal blastemal masses.

At the mid-fission stage, high levels of *Nco-Pax6A* transcripts are detected in broad bilateral superficial domains corresponding to the area of eye development and the formation of a dense cluster of sensory hair cells in the prostomium and pygidium. The internal domains of expression of this gene coincide with the localization of cells that give rise to the brain, suboesophageal ganglion, and ventral nerve cord ganglia. At the mid-fission stage, the other three genes (*Nco-Pax6B*, *Nco-Pax6C*, and *Nco-Pax6D*) are expressed de novo in the paratomy zone. All of them are active in cells of the developing ganglia, eyes, and prostomium, although their expression is weaker and more diffuse. Within the inner blastemal masses, the broadest diffuse expression is characteristic of *Nco-Pax6B*. It persists throughout the mid- and late stages of paratomy zone development. Furthermore, low levels of *Nco-Pax6B* transcripts are observed in the internal tissues of the newly developed cephalic end of the posterior zooid immediately after physical separation of new individuals, as well as in the internal tissues of new segments of the posterior end of the growing animal. Along with the growth of horizontal nerves, commissures, and segmental nerves in the developing cephalic and caudal ends, such a prolonged phase of broad diffuse expression suggests the involvement of *Nco-Pax6B* gene in the formation of nerve tracts and their topology [71,85].

At the late fission stage, the differential expression pattern of the identified genes is maintained. Within the new developing head, *Nco-Pax6A* expression begins to gradually disappear and becomes restricted to a few dorsal cells of the new brain and individual dorsal cells of the ventral nerve cord ganglia of the new head segments. Furthermore, at the posterior end of the animals, it is found not only in the terminal region of the ventral nerve cord of the youngest segments but also in individual cells localized dorsolaterally in the ventral nerve cord ganglia of the developed segments. Unlike *Nco-Pax6A*, expression of *Nco-Pax6B*, *Nco-Pax6C*, and *Nco-Pax6D* persists throughout the late fission stage. Interestingly, mRNA of *Nco-Pax6B* and *Nco-Pax6D* is shown in several anterior-dorsal and posterior cells of the developing brain, without signs of colocalization with *Nco-Pax6A* expression. The spatial expression pattern in the developing brain, as well as dorsolateral expression in the new ganglia of the ventral nerve cord, may be evidence of the involvement of *Nco-Pax6* genes in dorsoventral patterning and regionalization of the central nervous system in *N. communis* [1,7,29,32,40,41,42,43,45]. *Nco-Pax6C* expression in cells of the developing eyes, as well as *Nco-Pax6D* expression in cells surrounding the eyes, persists during the late fission stage and immediately after physical separation of individuals from each other, but soon disappears and is not detected in adult growing worms.

Thus, the expression pattern of all four *Pax6* homologs identified in the annelid *N. communis* correlates with remodeling and development of the nervous system, as well as the formation of eyes and sensory organs during asexual reproduction. Our results support the hypothesis of an evolutionarily conserved function of *Pax6* genes in the development of the eye and other sensory organs, as well as the central nervous system, among bilaterians, regardless of developmental trajectory. Differences in spatiotemporal expression patterns may be evidence of functional diversification of duplicated homologs. Unfortunately, genomic resources, as well as methods for functional gene analysis, are not yet available for *Nais communis*. Further development of approaches and future studies may help us answer many questions about the possible subfunctionalization and/or neofunctionalization of *Pax6* homologs in this animal.

## 5. Conclusions

In this work, we provide insights on the molecular aspects of remodeling, development, and patterning of the nervous system in asexually reproducing annelids. The spatial expression pattern of identified *Pax6* paralogs in the developing brain, as well as in the new ganglia of the ventral nerve cord, may be evidence of the involvement of *Pax6* genes in dorsoventral patterning and regionalization of the central nervous system in the annelid *N. communis*. Results of our study suggest the evolutionarily conserved role of *Pax6* genes in development and patterning of the nervous system, despite the variability and evolution of the developmental trajectories. Differences in the expression patterns of paralogs may indicate that these genes have diversified their functions.

## Data Availability

mRNA sequences of *Nco-pax6A*, *Nco-pax6B*, *Nco-pax6C*, and *Nco-pax6D* are deposited in GenBank with the accession numbers PX514181-PX514184, respectively.

## Supplementary Materials

The following supporting information can be downloaded at: https://www.mdpi.com/article/10.3390/biology14121704/s1, Table S1. Primer sequences used to clone fragments of the *Nco-pax6A*, *Nco-pax6B*, *Nco-pax6C*, and *Nco-pax6D* genes presented in the paper; Tex S1. Multiple alignment (FASTA format) of Pax6 proteins; Figure S1. Phylogenetic analysis of Nais communis Pax6 homologs. Bayesian consensus tree of the fragments contained both paired domain and homeodomain domain of metazoan Pax6 genes. Figure S2. In situ hybridization with the sense DIG-labeled riboprobe, a negative control.
