# Supplementary material for: Duplicated Pax6 Gene Expression During Nervous System Development in the Asexually Reproducing Annelid Nais communis
Source: Biology (Basel). 2025 Nov 29;14(12):1704. doi: 10.3390/biology14121704 (PMC12730286; doi:10.3390/biology14121704)
Supplement: Supplementary file 1 [file biology-14-01704-s001.zip › biology-3998885-supplementary.pdf]

Supplementary Materials

Table S1. Primer sequences used to clone fragments of the *Nco-pax6A*, *Nco-pax6B*, *Nco-pax6C*, and *Nco-pax6D* genes presented in the paper.

			Fragment used for WMISH	5′-RACE fragment	3′-RACE fragment
Nco-pax6A	forward	5’- GCAGGACAACATACCAAGTGTGTC -3’	1290 bp		1373 bp
	reverse	5’- CATTGGATGCGAGGCCAGTACTGTGAC -3’		1777 bp	
Nco-pax6B	forward	5’- CCTGACCACCGAGACTCATAAGC -3’	1423 bp		1460 bp
	reverse	5’- CCAGTAGTGTGACGTAGCGGTTCC -3’		1798 bp	
Nco-pax6C	forward	5’- GACATCTCGCGCATTGTTCAGGTG -3’	1217 bp		1628 bp
	reverse	5’- GGAAGTGACCAACCCAACGACAG -3’		1431 bp	
Nco-pax6D	forward	5’- CCGAGTGTGTCGTCTATCAACAG -3’	1170 bp		1455 bp
	reverse	5’- CACTTGAAGCGGTACAGAAACACC -3’		1718 bp	

Tex S1. Multiple alignment (FASTA format) of Pax6 proteins

>CBY05536.1 Pax4 transcription factor [Danio rerio]

GEGSVNQLGGVFLNGRPLPVYKRRLMIELATEGMRPCEISRILK--------------VS

NGCVSKILGRFQRTGAIGPKAVGGSRPRLLTPEVISIIVQHKRQNPTLFAWEIRQKLATE

RACRGDQVPSVSSINRILKKIHQDVDIA--------------------------------

---------------------------------------YPNEKH---------YHTE--

-HL---

---------------NVQSHFQQQMNTNLRRTAHRSRTAFTADQSGRLEKEFTCGLYPDL

LTREKLAEETNLSQDTIKVWFSNRRARMRRERKFEHNDCGI---

>AUN27664.1 PAX3/7 [Owenia fusiformis]

GQGRVNQLGGVFINGRPLPNHIRLKIVEMASQGVRPCVISRQLR--------------VS

HGCVSKILQRYQETGSIRPGVIGGSKPRVATPQVERKIEDYKKENPGIFSWEIRDRLLKD

GVCDKMNVPSVSSISRVLRSRHRSVEDDGNASDEND------------------------

---V----------------

-------------------------------------T-ITPD--------------HSI

DGI---

---L--

------RSEKTELDSDVDSDTECEPGLKLERKQRRSRTTFTAEQIEVLEKAFERTHYPDI

FTREELAQRTKLTEARVQVWFSNRRARWRKQVGSSQISALN---

>Pax6C [Nais communis]

GHSGVNQLGGIFINGRPLPDTTRQRIVAMAQSGARPCDISRIVQ-------------VS

NGCVSKILGRYYETGTFRPRNIGGSKPRVATRSVMERIATLKTECPSIFAWEIRDRLLAD

GFCTQQSLPSISSINRVLRSMMVTSNSNSSLT---------------------------

--------------------------------------FNDHPFLSTS----------

--------------------------GQARPSDLNFQRWTTLTP-------------S

TVCCHVTTTSS-HS------------LDPCLSRPPA--------------------NQ

S-------TLAT-----------------VNVAALEAANCGFINEHLHRNQ--------

-------------HHQLLTRLHSTAPGYRKKLQRNRTAFSQSQVDSLEREFMTTHYPDV

HCRERLATTLHLPEARLQVWFSNRRAKWRREERSLSRHRQF---

>BAA24516.1 Pax4 [Mus musculus]

GLSSVNQLGGLFVNGRPLPLDTRQQIVQLAIRGMRPCDISRSLK-------------VS

NGCVSKILGRYYRTGVLEPKCIGGSKPRLATPAVVARIAQLKDEYPALFAWEIQHQLCTE

GLCTQDKAPSVSSINRVLRALQEDQSLH-------------------------------

-------------------------------------WTQLRS--PAVLAPVLPSPH-

-SN--

--------------------------------------CG-----------------

----------------------APRGPHPGTSHRNRTIFSPGQAEALEKEFQRGQYPDS

VARGKLAAATSLPEDTVRVWFSNRRAKWRRQEKLKWEAQLP---

>JAA92591.1 Pax6B [Dendrocoelum lacteum]

GHSGINQLGGMFVNGRPLPDVTRQRIIELSQSGARPCDISRILQ-------------VS

NGCVSKILCRFYETGSIKPKAIGGSKPRVATNSVVRKVTVYKQESPSMFAWEIRDRLLQD

GVCNQDNIPSISSINRILRSLSNETNTNSTPFKP-------------------------

----------------PLNNCHQLSIVNPHNSSNQSHSQYNPINNSTTT----------

----NFN---------------LLHS-----PSSFMNNWPSPPTA---VFPHWYSQSGI

SSLCHSTL---------------FGYNGTNYYPPHFQDYTHLE------ESKT------

--------RLNSITNQREPSVK--------STVHSVNED-----TANIKMDSEKT-----

------SE-YNSDRESEVDKKYSNSCSKLTKKSQRSRTSFTNDQISLLEKEFDRTHYPDV

FSREKLSHKLKVAETRIQVWFSNRRAKWRREEKAEDNINSN---

>ABN09915.2 paired box 6A transcription factor [Helobdella sp. MS-2000]

GHSGVNQLGGMFVNGRPLPDTTRQKIVELAHNGARPCDISRILQ-------------VS

NGCVSKILGRYYESGSIRPRTIGGSKPRVATMSVVQRIAHYKKECPSIFAWEIRDRLLSE

EICSQENIPSVSSINRVLRNLSSSTPSTPNSCLEKDSSTTSATS---TTTL--QHSNIQQ

QQQQ---HLQQLPMQMMPLNNSSNNNAK---NSS-NNNNIYDVIATNNGHVFNCYDYKNA

PTTTTT------------------------MLENMADIWATSQPTYKTYTKNVFLNQQQ

HHHHHQNRHHHQHTNDGDDDEDGGFACADDNDDSKNFPYLDKLNNN--INDS-CDENDNE

STTTTTTLTLTTLNNTNGNKEYTDLHATPTTT---MTSSSAA------------TTLES

TATTTSSTTTASRDRETGSDVEKQVRMRLKRKLQRNRTSFSTQQIDSLEREFDKTHYPDV

FARERLAQKLELPEARIQVWFSNRRAKWRREEKFRVVPGHH---

>Pax6D [Nais communis]

GHSGVNQLGGTFVNGRPLPDTTRQRIIELAHSGARPCDISRILQ-------------VS

NGCVSKILGRYYESGSIRPRTIGGSKPRVATDDVVSRIAVYKQECPSIFAWEIRDRLLAE

EICNPENIPSVSSINRVLRNLSTTGDAQKSFLDG-------------------------

--------------------------------H-SGTMYVDRFGLFSSTGHATGG----

-------------------------------GVGNGTAWTRTSGVDN---SGWYSTPSA

VTS-------------------------SLS--PMSSSCPALS----------------

---------QSSVGSTTSQ-----------RNVHQLT-------HSTIVTKKE---H---

---TRVGTGSVDSSPAPNNDRDDLLRMRLKRKLQRNRTSFTTSQIDELEKEFEKTHYPDV

FARERLALKISLPEARIQVWFSNRRAKWRREEKLRCQQQLH---

>Pax6B [Nais communis]

XHSGVNQLGGVFVNGRPLPDSTRQRIVELAHSGARPCDISRILQ-------------VS

NGCVSKILGRYYETGSIRPRAIGGSKPRVATPDVVTRIAQYKRECPSIFAWEIRDRLLSE

GLCNHDNIPSVSSINRVLRNLTTETHKHGGGAGHFSI----------------------

-----------------------------------SGHVYDKFGCLFGS----------

----------------------------------AQSAWPPR-T--G---NTWYSAPPP

GQV-------------------------GS-----AAAAAAMFHGTEMAVAARAVGVAE

--------ALHGAGHLTQLHSTTSLATPP--SYSTSTSDRKSSSTNKRKHDEDLSCVSPP

SASSVLGHPHRQSSTSADTSTDEQMRLRLKRKLQRNRTSFNTQQVDDLEKEFEKTHYPDV

FARERLAQKLDLPEARIQVWFSNRRAKWRREEKLRSQQRRG---

>NP_524638.3 twin of eyeless, isoform A [Drosophila melanogaster]

GHSGINQLGGVYVNGRPLPDSTRQKIVELAHSGARPCDISRILQ-------------VS

NGCVSKILGRYYETGSIKPRAIGGSKPRVATTPVVQKIADYKRECPSIFAWEIRDRLLSE

QVCNSDNIPSVSSINRVLRNLASQKEQQAQ-----------------------------

--------------------------------Q-QNESVYEKLRMFNGQTGG-------

--------------------------------------W------------AWYPSNTT

TAH-------------------------LT--LPPAASV------------VTSPANLS

------------GQA---------------------DRDDVQKRELQFSVEVSHTNSH

D-----STSDGNSEHNSSGDEDSQMRLRLKRKLQRNRTSFSNEQIDSLEKEFERTHYPDV

FARERLADKIGLPEARIQVWFSNRRAKWRREEKMRTQRRSA---

>NP_524628.2 eyeless, isoform A [Drosophila melanogaster]

GHSGVNQLGGVFVGGRPLPDSTRQKIVELAHSGARPCDISRILQ--------------VS

NGCVSKILGRYYETGSIRPRAIGGSKPRVATAEVVSKISQYKRECPSIFAWEIRDRLLQE

NVCTNDNIPSVSSINRVLRNLAAQKEQQSTGSGSSSTSAGNSISAKVSVSIGGNVSNVAS

GSRGTLSSSTDLMQTATPLNSSESGGASNSGEGS-EQEAIYEKLRLLNTQHAAGPGP-LE

PARAAPLVGQSPNHLGTRSSHPQLVHGNHQALQQHQQQSWPPR-H-YS---GSWYPTSL-

SEI--------------------------PISSAPNIASVTAYASGPSLAHSLSPPNDIE

--------SLASIGHQ----------------------RNCPVATEDIHLKKELDGHQSD

ETGSGEGENSNGGASNIGNTEDDQARLILKRKLQRNRTSFTNDQIDSLEKEFERTHYPDV

FARERLAGKIGLPEARIQVWFSNRRAKWRREEKLRNQRRTP---

>CAA11367.1 Pax6 [Branchiostoma floridae]

GHSGVNQLGGVFVGGRPLPDSTRQKIVELAHQGARPCDISRLLQ--------------VS

NGCVSKILGRYYETGSIRPRAIGGSKPRVATPEVVAKIAQFKRECPSIFAWEIRDRLLSE

GICTNENIPSVSSINRVLRNLASGEKNTLQSL----------------------------

--------------------------------QS-ADPQMLEKLRLLNGN--A-------

---------------------------------------WPH-----P---GPWPYPPST

AGA--------------------------PPPQ---------------------------

---------------T----------------------NGNVTTK------KEGDGKLAS

QILTLH-GYQDQGDGSNDDSDEAQARLRLKRKLQRNRSSFTQEQIEALEKEFERTHYPDV

FARERLAAKIDLPEARIQVWFSNRRAKWRREEKLRNQRRSQ---

>AAH76068.1 Pax6b protein [Danio rerio]

SHSGVNQLGGVFVNGRPLPDSTRQKIVELAHSGARPCDISRILQ--------------VS

NGCVSKILGRYYETGSIRPRAIGGSKPRVATPEVVGKIAQYKRECPSIFAWEIRDRLLSE

GVCTNDNIPSVSSINRVLRNLASEKQQ---------------------------------

---------------------------------M-GADGMYDKLRMLNGQSGT-------

---------------------------------------WGT-----R---PGWYPGTSV

PGQ--------------------------PNQ----------------------------

--------------------------------------DGCQQQD------N--GGEN--

---------TNSISSNGEDSDETQMRLQLKRKLQRNRTSFTQEQIEALEKEFERTHYPDV

FARERLAAKIDLPEARIQVWFSMLGRPD---TALTNTYTGLPPM

>AAB36683.1 Pax6 [Xenopus laevis]

SHSGVNQLGGVFVNGRPLPDSTRQKIVELAHSGARPCDISRILQ--------------VS

NGCVSKILGRYYETGSIRPRAIGGSKPRVATPEVVNKIAHYKRECPSIFAWEIRDRLLSE

GVCTNDNIPSVSSINRVLRNLASDKQQ--------------------------------

--------------------------------M-GSEGMYDKLRMLNGQTAT-------

--------------------------------------WGS-----R---PGWYPGTSV

PGQ-------------------------PAQ---------------------------

-------------------------------------EGCQPQE------G--VGEN--

---------TNSISSNGEDSDEAQMRLQLKRKLQRNRTSFTQEQIEALEKEFERTHYPDV

FARERLAAKIDLPEARIQVWFSNRRAKWRREEKLRNQRRQA---

>AAH66722.1 Pax6a protein [Danio rerio]

SHSGVNQLGGVFVNGRPLPDSTRQKIVELAHSGARPCDISRILQ-------------VS

NGCVSKILGRYYETGSIRPRAIGGSKPRVATPEVVGKIAQYKRECPSIFAWEIRDRLLSE

GVCTNDNIPSVSSINRVLRNLASEKQQ--------------------------------

--------------------------------M-GADGMYEKLRMLNGQTGT-------

--------------------------------------WGT-----R---PGWYPGTSV

PGQ-------------------------PNQ---------------------------

-------------------------------------DGCQQSD------G--GGEN--

---------TNSISSNGEDSDETQMRLQLKRKLQRNRTSFTQEQIEALEKEFERTHYPDV

FARERLAAKIDLPEARIQVWFSNRRAKWRREEKLRNQRRQA---

>XP_024151553.1 paired box protein Pax-6b isoform X1 [Oryzias melastigma]

GHSGVNQLGGVFVNGRPLPDSTRQKIVELAHSGARPCDISRILQTHDE--VQVLDSEKVS

NGCVSKILGRYYETGSIRPRAIGGSKPRVATPEVVAKIAQYKRECPSIFAWEIRDRLLSE

GICTNDNIPSVSSINRVLRNLASEKQQ--------------------------------

--------------------------------M-GADGMYDKLRMLNGQTGT-------

--------------------------------------WGT-----R---PGWYPGTSV

PGQ-------------------------PNQ---------------------------

-------------------------------------EGCQQQD------G--AGEN--

---------TNSISSNGEDSEETQMRLQLKRKLQRNRTSFTQEQIEALEKEFERTHYPDV

FARERLAAKIDLPEARIQVWFSNRRAKWRREEKLRNQRRQA---

>CAA04395.1 Pax6 [Oryzias latipes]

SHSGVNQLGGVFVNGRPLPDSTRQKIVELAHSGARPCDISRILQ-------------VS

NGCVSKILGRYYETGSIRPRAIGGSKPRVATPEVVAKIAQYKRECPSIFAWEIRDRLLSE

GICTNDNIPSVSSINRVLRNLASEKQQ--------------------------------

--------------------------------M-GADGMYDKLRMLNGQTGT-------

--------------------------------------WGT-----R---PGWYPGTSV

PGQ-------------------------PNQ---------------------------

--------------------------------------DGCQQQD------G--AGEN--

---------TNSISSNGEDSEETQMRLQLKRKLQRNRTSFTQEQIEALEKEFERTHYPDV

FARERLAAKIDLPEARIQVWFSNRRAKWRREEKLRNQRRQA---

>BAA23004.1 PAX6 protein [Gallus gallus]

SHSGVNQLGGVFVNGRPLPDSTRQKIVELAHSGARPCDISRILQTHADAKVQVLDNQNVS

NGCVSKILGRYYETGSIRPRAIGGSKPRVATPEVVSKIAQYKRECPSIFAWEIRDRLLSE

GVCTNDNIPSVSSINRVLRNLASEKQQ---------------------------------

---------------------------------M-GADGMYDKLRMLNGQTGT-------

---------------------------------------WGT-----R---PGWYPGTSV

PGQ--------------------------PAQ----------------------------

--------------------------------------DGCPQQE------G--GGEN--

---------TNSISSNGEDSDEAQMRLQLKRKLQRNRTSFTQEQIEALEKEFERTHYPDV

FARERLAAKIDLPEARIQVWFSNRRAKWRREEKLRNQRRQA---

>NP_001006763.1 paired box protein Pax-6 [Xenopus tropicalis]

SHSGVNQLGGVFVNGRPLPDSTRQKIVELAHSGARPCDISRILQ--------------VS

NGCVSKILGRYYETGSIRPRAIGGSKPRVATPEVVSKIAQYKRECPSIFAWEIRDRLLSE

GVCTNDNIPSVSSINRVLRNLASEKQQ---------------------------------

---------------------------------M-GADGMYDKLRMLNGQTGT-------

---------------------------------------WGT-----R---PGWYPGTSV

PGQ--------------------------PAQ----------------------------

--------------------------------------DGCQPQE------GGGGGEN--

---------TNSISSNGEDSDEAQMRLQLKRKLQRNRTSFTQEQIEALEKEFERTHYPDV

FARERLAAKIDLPEARIQVWFSNRRAKWRREEKLRNQRRQA---

>AAH36957.1 Pax6 protein [Mus musculus]

SHSGVNQLGGVFVNGRPLPDSTRQKIVELAHSGARPCDISRILQ--------------VS

NGCVSKILGRYYETGSIRPRAIGGSKPRVATPEVVSKIAQYKRECPSIFAWEIRDRLLSE

GVCTNDNIPSVSSINRVLRNLASEKQQ---------------------------------

---------------------------------M-GADGMYDKLRMLNGQTGS-------

---------------------------------------WGT-----R---PGWYPGTSV

PGQ--------------------------PTQ----------------------------

--------------------------------------DGCQQQE------GG--GEN--

---------TNSISSNGEDSDEAQMRLQLKRKLQRNRTSFTQEQIEALEKEFERTHYPDV

FARERLAAKIDLPEARIQVWFSNRRAKWRREEKLRNQRRQA---

>NP_000271.1 paired box protein Pax-6 isoform a [Homo sapiens]

SHSGVNQLGGVFVNGRPLPDSTRQKIVELAHSGARPCDISRILQ--------------VS

NGCVSKILGRYYETGSIRPRAIGGSKPRVATPEVVSKIAQYKRECPSIFAWEIRDRLLSE

GVCTNDNIPSVSSINRVLRNLASEKQQ---------------------------------

---------------------------------M-GADGMYDKLRMLNGQTGS-------

---------------------------------------WGT-----R---PGWYPGTSV

PGQ--------------------------PTQ----------------------------

--------------------------------------DGCQQQE------GG--GEN--

---------TNSISSNGEDSDEAQMRLQLKRKLQRNRTSFTQEQIEALEKEFERTHYPDV

FARERLAAKIDLPEARIQVWFSNRRAKWRREEKLRNQRRQA---

>AAI28742.1 Paired box 6 [Rattus norvegicus]

SHSGVNQLGGVFVNGRPLPDSTRQKIVELAHSGARPCDISRILQ--------------VS

NGCVSKILGRYYETGSIRPRAIGGSKPRVATPEVVSKIAQYKRECPSIFAWEIRDRLLSE

GVCTNDNIPSVSSINRVLRNLASEKQQ---------------------------------

---------------------------------M-GADGMYDKLRMLNGQTGS-------

---------------------------------------WGT-----R---PGWYPGTSV

PGQ--------------------------PTQ----------------------------

--------------------------------------DGCQQQE------GQ--GEN--

---------TNSISSNGEDSDEAQMRLQLKRKLQRNRTSFTQEQIEALEKEFERTHYPDV

FARERLAAKIDLPEARIQVWFSNRRAKWRREEKLRNQRRQA---

>Pax6A [Nais communis]

GHSGVNQLGGMFVNGRPLPDTTRQRIVELAHSGARPCDISRILQ--------------VS

NGCVSKILGRYYESGSIRPRTIGGSKPRVATNDVVRRIAQYKRECPSIFAWEIRDRLLAE

EACTQDNIPSVSSINRVLRNLTTDTPQYSIR-----------------------------

------------------------------------GSINYDKCGATG----NTPTR-LT

PGGGSLF----------------------------SGETWHR-----T---NPWYAAASA

AAP--------------------------NNQARIGTSNT-------------------G

--------SSTG--QVPNNN----------SNYHPPTSTSSCVTSNNTS---QKDHHSSE

SVTRKQHCSDGVSPAHNGAESDEQLRMRLKRKLQRNRTSFTTQQIDDLEKEFEKTHYPDV

FTRERLAQKLDLPEARIQVWFSNRRAKWRREEKMHGHNPRR---

>JAA92592.1 Pax6A, partial [Dendrocoelum lacteum]

GHSGVNQLGGMFVNGRPLPDSTRQRIVELAHSGARPCDISRILQ--------------VS

NGCVSKILCRYYETGSIRPKAIGGSKPRVATSSVVAKIAGYKRECPSIFSWEIRDRLLQE

GVCNQDNIPSVSSINRVLRSLSNENHRQLAA-----------------------------

------------------------------------ATGMYDKL----------------

----SLL----------------------------SGQPWSSAAA-AH---AAWYSSAA-

-AA--------------------------HGYASSTFPNCG----------------TYG

--------SLTGIGIINGMNSAHAVASMNQSNSS-INNYQLQTSAESEK---PKSAGSVS

QSESNASSENGHDYMSGLKGENDDMRVKLKRKLQRNRTSFSTDQLDSLEKEFERTHYPDV

FAREKLADKITLPEARIQVWFSNRRAKWRREEKLRRQRQNL---

>ARJ36945.1 pax6 [Membranipora membranacea]

GHSGVNQLGGVFVNGRPLPESTRRKIVELAHSGARPCDISRILQ--------------VS

NGCVSKILGRYYETGSIRPRAIGGSKPRVATAEVVAKIAQYKRECPSIFAWEIRDRLLQE

QVCNQDNIPSVSSINRVLRNLASENQKQMA------------------------------

------------------------------------AGSMYPA-----------------

------L----------------------------YNGQWGRP----T----GWYHPNNP

AAL--------------------------SAQYQQTIPPPP-------------------

----------------------------Q---------D--------IK---PKTAD---

-----DINDNNSASGESGGGDEDNMRLRLKRKLQRNRTSFTNAQIESLEKEFERTHYPDV

FARERLAKEIDLPEARIQVWFSNRRAKWRREEKLRNQRRDQ---

>KAF6020944.1 PAX6 [Bugula neritina]

GHSGVNQLGGVFVNGRPLPESTRRKIVELAHSGARPCDISRILQ--------------VS

NGCVSKILGRYYETGSIRPRAIGGSKPRVATAEVVNKIAQYKRECPSIFAWEIRDRLLQE

NVCNQDNIPSVSSINRVLRNLASENQKQMT------------------------------

------------------------------------AGSMYPA-----------------

------L----------------------------YGGQWGRP----A---ASWYHPNNA

ATI--------------------------SAQYPPSLPPTQ-------------------

----------------------------P---------D--------MK---PKVND---

-----DLGDNNSTGGESQGGDDDNMRLRLKRKLQRNRTSFTNAQIESLEKEFERTHYPDV

FARERLAKEIDLPEARIQVWFSNRRAKWRREEKLRNQRREP---

>AUN27670.1 PAX6 [Lineus ruber]

RHSGVNQLGGVFVNGRPLPDSTRQRIVELAHSGARPCDISRILQ--------------VS

NGCVSKILGRYYETGSIRPRAIGGSKPRVATPEVVGKIAHYKRECPSIFAWEIRDRLLSD

AVCNQDNIPSVSSINRVLRNLASENQKQLG------------------------------

------------------------------------QSSMYDKL----------------

----GLL----------------------------NGQAWPR-----P---NPWYAPNTH

PAM--------------------------TGLTAHHPQYPP-------------------

----------------------------PQPQPPPISPTKKE-----------SDGH---

-----SSADSHSGDTPNGNESEEQMRIRLKRKLQRNRTSFTNAQIEALEKEFANLTHEKR

I---

>ABN09916.2 paired box 6B transcription factor, partial [Helobdella sp. MS-2000]

XXSGVNQLGGVFVNGRPLPDSTRQRIVELAHSGARPCDISRILQ--------------VS

NGCVSKILGRYYETGSIRPRAIGGSKPRVATPEVVNKIAQYKGECPSIFAWEIRDRLLSE

CLCTQENIPSVSSINRVLRNLTSETHKSQLN-----------------------------

------------------------------------QGQMYEKF----------------

----SLF----------------------------GGQAWHRA----T---NPWYSSTA-

APM--------------------------HPISMATQHQLT-------------------

----------------------------N-S--AFYNSFEKKGLLSKRK---PEEDA---

-----LTSNESCDSSPRANETDEQMRMRLKRKLQRNRTSFTTQQIEDLEKEFEKTHYPDV

FARERLAQKLDLPEARIQVWFSNRRAKWRREEKLRNQRRDV---

>AUN27663.1 PAX6 [Owenia fusiformis]

GHSGVNQLGGVFVNGRPLPDSTRQRIVELAHSGARPCDISRILQ--------------VS

NGCVSKILGRYYETGSIRPRAIGGSKPRVATNEVCAKVAQYKRECPSIFAWEIRDRLLSD

NVCNQDNIPSVSSINRVLRNLASENQKVLG------------------------------

------------------------------------QGSMYDKL----------------

----GLL----------------------------NGQVWPR-----P---NPWYSTPQ-

ATM--------------------------PGLGMTHSPYGQ-------------------

----------------------------S-HQISPPIQDKKEGILENT-------SA---

-----ESPGSHTGGESANGEQDDQMRIRLKRKLQRNRTSFTNAQIEALEKEFERTHYPDV

FARERLAQKIDLPEARIQVWFSNRRAKWRREEKLRNQRRDV---

>WAR02137.1 PAX6-like protein [Mya arenaria]

----------MFVNGRPLPDSTRQRIVELAHSGARPCDISRILQ--------------VS

NGCVSKILGRYYETGSIRPRAIGGSKPRVATNDVCSHVAQYKRECPSIFAWEIRDRLLSD

GVCNQENIPSVSSINRVLRNLATENQKVMG------------------------------

------------------------------------QGSMYDKL----------------

----GLL----------------------------NGQAWAR-----P---NPWY--PN-

MGM--------------------------HGISAPTY--HQ-------------------

----------------------------P-PNAHLSMEKKH----NDA--------N---

-----SSSDSNHSDGAKGSENDEQMRMRLKRKLQRNRTSFTASQIEALEKEFERTHYPDV

FARERLAQKIDLPEARIQVWFSNRRAKWRREEKLRNQRREP---

>ALS19770.1 pax6 [Novocrania anomala]

GHSGVNQLGGVFVNGRPLPDSTRQRIVELAHSGARPCDISRILQ--------------VS

NGCVSKILGRYYETGSIRPRAIGGSKPRVATPEVVTKIAVYKRECPSIFAWEIRDRLLSD

GICTQDNIPSVSSINRVLRNLASDNQKPML----------------------------

------------------------------------PNAMYDKL----------------

----GMQ---------------------------NGLAWPR-----P---NPWYA-PN-

PGL-------------------------TGQYNPPPPPSQ------------------

---------------------------T-S----VGDVKKE-----E--------S---

-----NTEASSPGGEAQTNESDEQMRIRLKRKLQRNRTSFTNAQIESLEKEFERTHYPDV

FARERLAQKIDLPEARIQVWFSNRRAKWRREEKLRNQRRDA---

>AAM74161.1 Pax-6 protein [Euprymna scolopes]

GHSGVNQLGGVFVNGRPLPDSTRQRIVELAHSGARPCDISRILQ-------------VS

NGCVSKILGRYYETGSIRPRAIGGSKPRVATPEVVQKIAQFKRECPSIFAWEIRDRLLSE

GVCTQDNIPSVSSINRVLRNLASENQKVLGQ----------------------------

------------------------------------GTTMYDKL----------------

----GLL---------------------------NGQAWPR-----P---NPWYA-PN-

ASM-------------------------AGLSAPSSYTQ-------------------

-----------------------------PNAPSAVSGKKEM--ENL--------S---

-----TSSDNGQGDGSQNGETDEQMRIRLKRKLQRNRTSFTAAQIEALEKEFERTHYPDV

FARERLAQKIDLPEARIQVWFSNRRAKWRREEKLRNQRREA---

>CAJ40659.1 Pax6 protein [Platynereis dumerilii]

GHSGVNQLGGVFVNGRPLPDSTRQRIVELAHSGARPCDISRILQ-------------VS

NGCVSKILGRYYETGSIRPRAIGGSKPRVATPEVVNKVAQYKRECPSIFAWEIRDRLLSE

GVCNQDDIPSVSSINRVLRNLASETQKTTLS----------------------------

------------------------------------QNPMYDKL----------------

----GFL---------------------------NGQAWPR-----T---NPWYA-PN-

APM-------------------------HGLSMSPPYQ--------------------

---------------------------P-PNPPIPPPEKKD------------------

------SASTGSSGSDNPNCDEEQMRMRLKRKLQRNRTSFTNAQIEALEKEFERTHYPDV

FTRERLAKKFDIDETRIQVWFSNRRAKWRREEKLRQQRREA---

>AMB21744.1 Pax6 [Ridgeia piscesae]

GHSGVNQLGGVFVNGRPLPDSTRQRIVELAHSGARPCDISRILQ-------------VS

NGCVSKILGRYYETGSIRPRAIGGSKPRVTTPEVVQKIAQYKRECPSIFAWEIRDRLLSE

AVCNQDNIPSVSSINRVLRNLTSDTQKSPLC----------------------------

------------------------------------QSPIYDKF----------------

----GLL---------------------------NGQSWPR-----P---SPWYT-PG-

APM-------------------------HGIGMTTPYPSQ------------------

---------------------------P-RSPTHPIPEKKEI--T-------------

-----NCSSQGSNGSAHNGETDEQMRMRLKRKLQRNRTSFTNAQIEALEKEFEKTHYPDV

FARERLAQKLDLPEARIQVWFSNRRAKWRREEKLRNQRRDV---

Figure S1. Phylogenetic analysis of *Nais communis Pax6* homologs. Bayesian consensus tree of the fragments contained both paired domain and homeodomain domain of metazoan *Pax6* genes. Protein names, except for Nco-Pax6, include GenBank accession numbers.

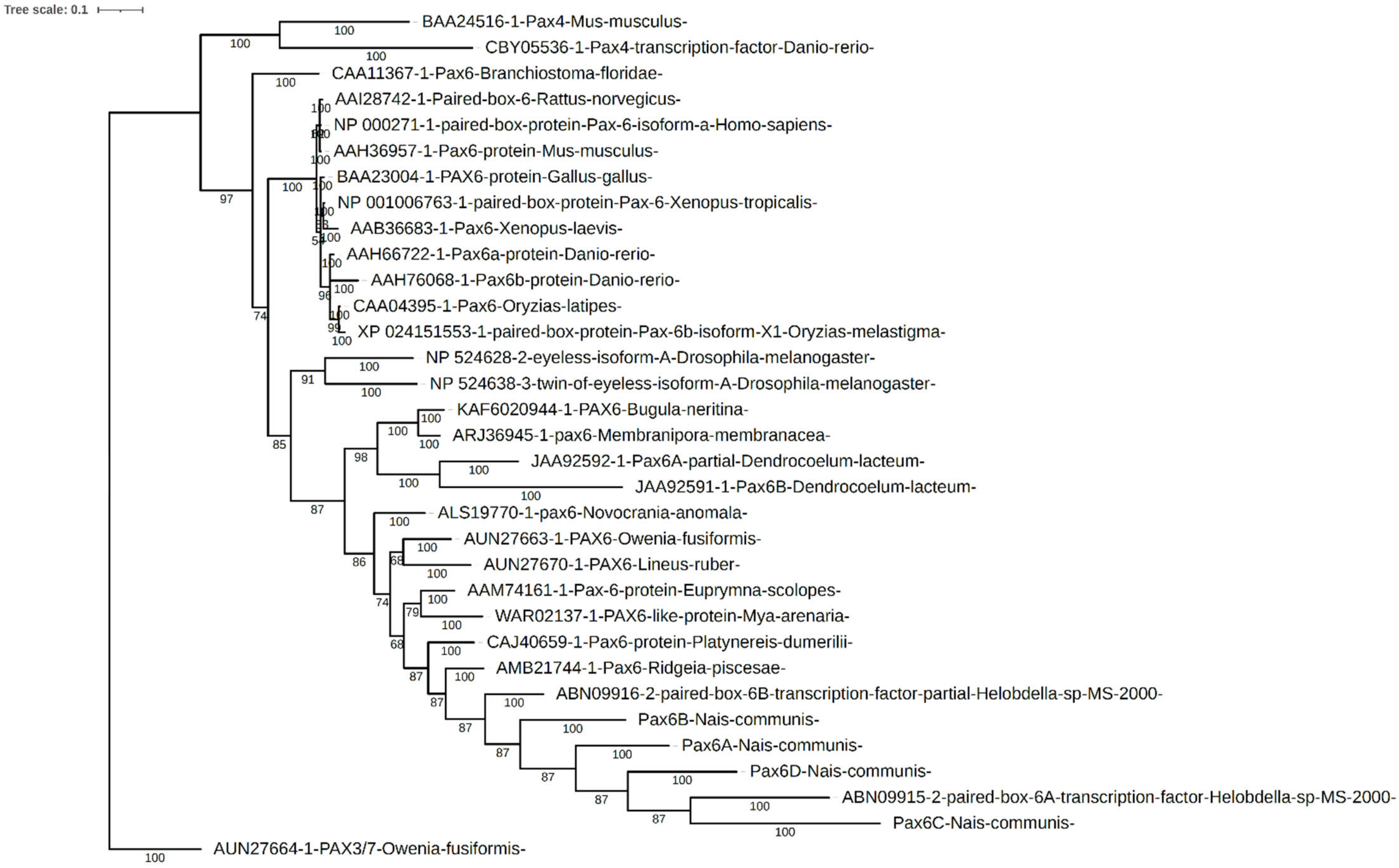

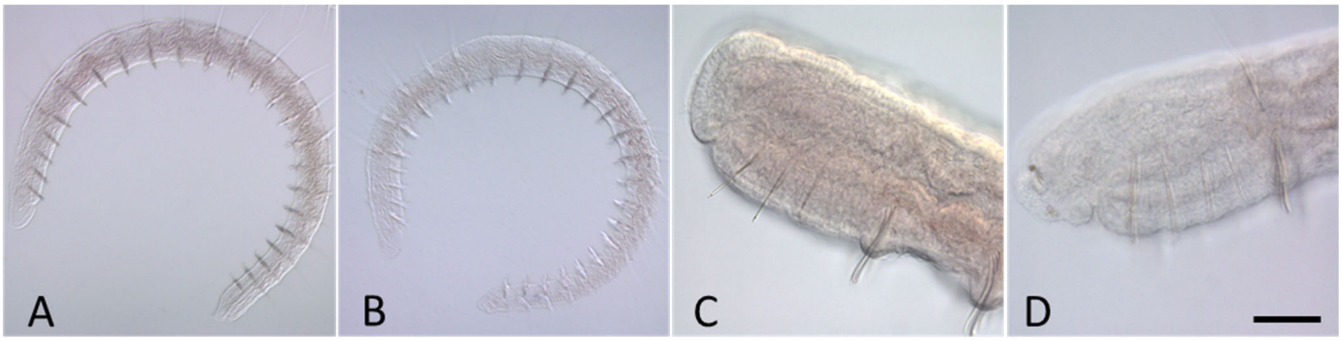

Figure S2. *In situ* hybridization with the sense DIG-labeled riboprobe, a negative control. All animals are oriented anterior to the left. Lateral view for all panels. (**A**) *Nco-pax6A*. (**B**) *Nco-pax6B*. (**C**) *Nco-pax6C*. (**D**) *Nco-pax6D*. Scale bar, 40 μm for **A**, **B**. Scale bar, 140 μm for **C**, **D**.
